# Negative regulatory responses to metabolically triggered inflammation impair renal epithelial immunity in diabetes mellitus

**DOI:** 10.1007/s00109-012-0969-x

**Published:** 2012-11-14

**Authors:** Nelson K. F. Chen, Tsung Wen Chong, Hwai-Liang Loh, Kiat Hon Lim, Valerie H. L. Gan, Marian Wang, Oi Lian Kon

**Affiliations:** 1 Division of Medical Sciences, Humphrey Oei Institute of Cancer Research, National Cancer Centre, Singapore, 169610 Singapore; 2 Urology Centre, Singapore General Hospital, Singapore, 169608 Singapore; 3 Pathology Department, Singapore General Hospital, Singapore, 169608 Singapore; 4 Department of Biochemistry, Yong Loo Lin School of Medicine, National University of Singapore, 8 Medical Drive, Singapore, 117597 Singapore; 5 Present Address: Centre for Forensic Medicine, Health Sciences Authority, Singapore, 169078 Singapore

**Keywords:** Diabetes mellitus, Inflammation, Immune homeostasis, Renal epithelium, Bacterial infection, Cytomegalovirus

## Abstract

Diabetes mellitus is characterized by chronic inflammation and increased risk of infections, particularly of tissues exposed to the external environment. However, the causal molecular mechanisms that affect immune cells and their functions in diabetes are unclear. Here we show, by transcript and protein analyses, signatures of glucose-induced tissue damage, chronic inflammation, oxidative stress, and dysregulated expression of multiple inflammation- and immunity-related molecules in diabetic kidneys compared with non-diabetic controls. Abnormal signaling involving cytokines, G-protein coupled receptors, protein kinase C isoforms, mitogen-activated protein kinases, nuclear factor-κB (NFκB), and Toll-like receptors (TLR) were evident. These were accompanied by overexpression of negative regulators of NFκB, TLR, and other proinflammatory pathways, e.g., A20, SOCS1, IRAK-M, IκBα, Triad3A, Tollip, SIGIRR, and ST2L. Anti-inflammatory and immunomodulatory molecules, e.g., IL-10, IL-4, and TSLP that favor T_H_2 responses were strongly induced. These molecular indicators of immune dysfunction led us to detect the cryptic presence of bacteria and human cytomegalovirus in more than one third of kidneys of diabetic subjects but none in non-diabetic kidneys. Similar signaling abnormalities could be induced in primary human renal tubular epithelial (but not mesangial) cell cultures exposed to high glucose, proinflammatory cytokines and methylglyoxal, and were reversed by combined pharmacological treatment with an antioxidant and a PKC inhibitor. Our results suggest that diabetes impairs epithelial immunity as a consequence of chronic and inappropriate activation of counter-regulatory immune responses, which are otherwise physiological protective mechanisms against inflammation. The immune abnormalities and cryptic renal infections described here may contribute to progression of diabetic nephropathy.

**Electronic supplementary material:**

The online version of this article (doi:10.1007/s00109-012-0969-x) contains supplementary material, which is available to authorized users.

## Introduction

Many studies have shown infection to be a major cause of death among diabetics [1–5]. A prospective 15-year study revealed a 41-fold higher risk of death from infections compared with the referent population [5]. Host-specific factors have been implicated as contributing to abnormalities of innate and adaptive immunity [6–9]. Abnormalities of innate immunity mediated by leukocytes, macrophages, natural killer, and dendritic cells have been observed in diabetes [7–9]. Disordered cell-mediated immunity in diabetes is reflected in reduced leukocyte adherence, chemotaxis and phagocytosis, impaired oxidative burst and intracellular bactericidal activities, and diminished production of antimicrobial cytokines [7, 10–13]. Diabetes also impairs adaptive immunity via the cell-mediated arm [14, 15] and humoral immunity [7]. Despite evidence of diverse alterations of immune function in vitro, their relevance to the risk of infections among diabetic patients in vivo is controversial [16–18]. Nonetheless, indubitable evidence correlates good glycemic control with improved immune function and lower infection rates [1, 14, 19].

Here, we describe mechanisms that impair epithelial immunity in kidneys in human diabetes. We hypothesized that cross-talk between immune cells and resident tissue cells could be key in maintaining tissue immune homeostasis and mounting appropriate immune responses. Epithelial cells can detect microbial infections and are indispensable in “coaching” the behavior of immune cells [20, 21].

We chose kidney as a model tissue because urinary tract infection is common among diabetics and diabetic end-stage renal disease remains a major clinical challenge. We analyzed histologically normal, fresh human kidney tissues procured from non-diabetic and diabetic subjects who had clinically normal renal function. The use of non-nephropathic kidney tissues allowed us to document molecular perturbations that precede clinical complications of chronic diabetes, unconfounded by renal parenchymal disease. As diabetes-associated target tissue injury occurs before loss of clinical function [22], documenting pre-clinical molecular dysregulations may enable more effective approaches to prevent and treat diabetes-induced renal dysfunction.

Our results suggest that diabetes and hyperglycemia per se impair renal epithelial immunity as a consequence of inappropriately activated negative counter-regulatory responses, which are physiological protective mechanisms against chronic inflammation. The immune abnormalities and cryptic bacterial and viral infections we discovered in the kidneys of diabetic subjects may predispose to diabetic renal disease.

## Materials and methods

### Human kidney tissues

DNA, RNA, and protein were extracted from tumor-distant tissues of kidneys surgically removed for renal carcinoma. All patients received no anticancer treatment before nephrectomy. Bacteria and human CMV were detected in DNA from fresh and formalin-fixed paraffin-embedded (FFPE) kidney tissues. Table S1 in the Electronic supplementary material (ESM) summarizes the clinical profiles of all patients in this study which was approved by the SingHealth Institutional Review Board.

### DNA, RNA, and protein isolation from human kidney

Kidney tissues, flash frozen in liquid nitrogen, were verified to be cancer free and to have no diagnostic features specific for diabetic nephropathy by histological examination. None of the patients had clinical evidence of urinary infection or significant albuminuria. Frozen tissues were diced in AllProtect tissue reagent (Qiagen), immersed in tissue lysis buffer for DNA, RNA, or protein isolation and disrupted in Tissuelyser LT (Qiagen). DNA, RNA, or proteins were purified from tissue lysates using the appropriate kits and protocols (Qiagen). Membrane proteins were isolated with Q proteome cell compartment kit (Qiagen). On-column digestion of DNA was performed during RNA isolation using RNase-free DNase (Qiagen).

### Antibodies and reagents

Suppliers of antibodies, reagents, and kits were: antibodies against IRAK isoforms, MyD88, RIG-1, A20, SOCS1, NIK and IκBα, NFκB p65, p38 MAPK, JNK, Akt, and their phosphorylated proteins (Cell Signaling Technology); antibodies against actin, Triad3A, TRAF6, protein kinase C (PKC)-βII, Sod2, Nox4, and goat IgG-HRP (Santa Cruz Biotechnology); antibodies against TLR4 and phosphorylated PKC-βII (Abcam); clinical grade CellGro® GMP TNF-α, IL-6, and IL-1β (CellGenix GmbH); methylglyoxal, lipopolysaccharide (LPS) from *Escherichia coli* 055:B5, lipoteichoic acid from *Staphylococcus aureus*, diphenyleneiodonium chloride (DPI), and Gö6983 (Sigma-Aldrich); poly(I:C) (Pharmacia Biotech); iScript™ cDNA synthesis kit (Bio-Rad); protease inhibitor cocktail (Roche), and western blot reagents (Amersham ECL™ blocking agent, Advance western blotting detection kit, and mouse- and rabbit IgG-HRP antibodies) were from GE Healthcare.

### Cell culture

Clonetics® human primary renal tubular epithelial cells (RPTEC) and mesangial cells (NHMC), purchased from Lonza BioSciences, were cultured in REGM™ Renal Epithelial Cell Growth Medium and NHMC in MsGM™ Mesangial Cell Growth Medium, respectively. Cells were subcultured with ReagentPack™ Subculture Reagents. For all experiments, 2.5 × 10^4^ RPTEC/well or 3.5 × 10^4^ NHMC/well were seeded in a six-well plate at 37 °C, 5 % CO_2_ and cultured for 3 days before experiments commenced. Cells having the same experimental treatment were lysed directly in situ and pooled for total RNA and protein isolation. Endotoxin levels of culture media were assayed with an endotoxin detection kit (Lonza Biosciences).

### Western blot analysis

Protein concentrations of fresh frozen kidney tissue lysates (*n* = 8 each for diabetic and non-diabetic samples) and primary renal cell lysates were quantitated using the bicinchoninic acid method (Thermo Scientific). Lysates were resolved on SDS-PAGE and transferred onto PVDF membranes using Trans-blot Semidry (Bio-Rad). Immunoblotting was performed using individual primary antibodies, goat-, mouse- or rabbit IgG-HRP secondary antibodies, proprietary blocking, and detection reagents stated above.

### Quantitative RT-PCR

Transcript levels of 90 genes in fresh frozen human kidney tissues and primary renal cell samples were assayed with custom 96-gene RT^2^ Profiler™ PCR arrays (SABiosciences, Qiagen). Six diabetic kidneys positive for both bacteria and CMV were compared with six non-diabetic kidneys that were negative for both. Briefly, first-strand cDNA was generated with RT^2^ First Strand Kit. Quantitative PCR was performed using RT^2^ quantitative polymerase chain reaction (qPCR) master mix and proprietary primer pairs for each of the 90 genes (Bio-Rad CX96 thermal cycler). The transcript level of each gene was normalized with reference to *GAPDH*, *ACTB*, and *RPL13A*. Criteria for efficiency of reverse transcription and PCR, and genomic DNA contamination met the standards specified by the supplier.

### Quantitation of cytokines

Levels of 20 cytokines in fresh frozen kidney tissue lysates (*n* = 9 each for diabetic and non-diabetic samples) and primary renal cell lysates were quantitated by a sandwich-ELISA method using a custom Quantibody® Human Cytokine Array (RayBiotech, Inc.). Fluorescence signals acquired with Axon GenePix (Molecular Devices) were analyzed with RayBio® Q Analyzer (RayBiotech, Inc.)

### Detection of microbial DNA in kidneys

FFPE kidney tissues of patients of known diabetes status were sectioned for DNA isolation using QIAamp DNA FFPE tissue kit (Qiagen). Sections from the surface of tissue blocks were not used. Quantitative PCR using GoTaq® qPCR master mix (Promega) and primer pairs that amplify a 1.3-kb DNA sequence specific to bacteria [23] was performed on 50 ng DNA isolated from FFPE or fresh frozen kidney tissues. The amount of bacterial DNA in the sample was determined from a standard curve generated from unrelated human DNA spiked with known amounts of bacterial DNA isolated from *Lactobacillus* sp. and *E. coli*. CMV DNA was detected using Artus® CMV RG PCR kit (Qiagen). Table S1 in the ESM lists the source of tissues for microbial DNA analysis.

### Data analysis

All data were expressed as mean ± SEM. Statistical analyses (GraphPad Prism®) between groups were performed with parametric, unpaired Student’s *t* test. Welch’s correction was included if the variance was unequal. Linear correlation was performed to establish association.

All genes and proteins studied are listed in functional groups in Table S2 in the ESM.

## Results

### Kidney tissues express molecular signatures of diabetes

Fresh frozen tumor-free kidney tissues were obtained from nine type 2 diabetic and nine non-diabetic subjects of comparable age, body mass index, and gender distribution (Table S1 in the ESM). The diabetes status of each patient was identified by the most recent glycated hemoglobin (HbA_1c_) level and/or a record of current treatment for diabetes. All patients had unimpaired renal function (serum creatinine, ≤110 μM (males) or ≤85 μM (females)).

**Fig. 1:**
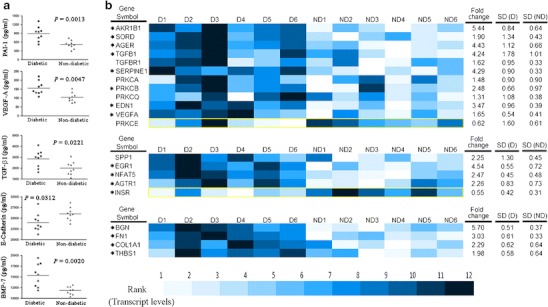
Tumor-free tissues from nephrectomized diabetic kidneys show molecular features of diabetes mellitus. **a** Protein lysates of histopathologically qualified tumor-free renal cortical tissues from diabetic and non-diabetic patients were assayed for the proteins indicated using a multiplex ELISA-based array (RayBiotech Inc.). The specific protein concentration in each kidney lysate was assayed in quadruplicate and quantitated from a standard curve, built into the multiplexed assay, using markers of known concentrations. All diabetic samples (*n* = 9) were positive for HCMV, bacteria or both. All non-diabetic samples (*n* = 9) were negative for HCMV and bacteria. **b** Tumor-free renal cortical tissues from diabetic and non-diabetic patients were homogenized for DNA, RNA, and protein isolation. RNA integrity and quality were verified (Bioanalyzer, Agilent) before qPCR assay. Shown are the results of qPCR array (SABiosciences, Qiagen) that assayed for 21 gene transcript levels using proprietary primers. The transcript level of each gene in each kidney sample was inter-ranked and presented as a pre-assigned color dendogram (*D* diabetic patients 1–6, *ND* non-diabetic patients 1–6). Each diabetic sample had evidence of both HCMV and bacterial infection while all non-diabetic samples were negative for both HCMV and bacteria. *Yellow-lined borders* highlight the only downregulated gene in each category. Fold change (D/ND) for each gene was calculated using the average transcript levels of all subjects in a group, which is accompanied by standard deviation. (*SD*)*. *P* ≤ 0.05, comparison between diabetic and non-diabetic is significant.

By quantitative RT-PCR, immunoblotting, and antibody arrays, diabetic renal tissues showed increased activity of the polyol and hexosamine pathways, PKC signaling, and increased expression of *AGER* compared with non-diabetic controls (Fig. 1) Other known abnormalities detected were increased expression of *NFAT5, EGR1, SPP1*, *INSR*, and *AGTR1*. Increased transcription of extracellular matrix protein genes, *BGN*, *FN1*, *COL1A1*, and *THBS1* coupled with decreased expression of E-cadherin in diabetic kidneys was consistent with epithelial–mesenchymal transition which has been implicated in the pathogenesis of diabetic nephropathy [24].

These data supported the use of tumor-free kidney tissues for studying tissue effects of diabetes unconfounded by renal parenchymal disease. Transcript levels of *AKR1B1*, *SORD*, *AGER*, *TGFB1*, *PAI1*, *PRKCB*, *EDN1*, *VEGFA*, *BGN*, *FN1*, *COL1A1*, *THBS1*, *EGR1*, and *NFAT5*, all implicated in diabetes complications, correlated significantly with HbA_1c_ levels of diabetic subjects (Fig. S1 in the ESM), confirming molecular effects of hyperglycemia on renal tissue even before functional impairment.

### Non-nephropathic kidneys of diabetic patients are inflamed

**Fig. 2:**
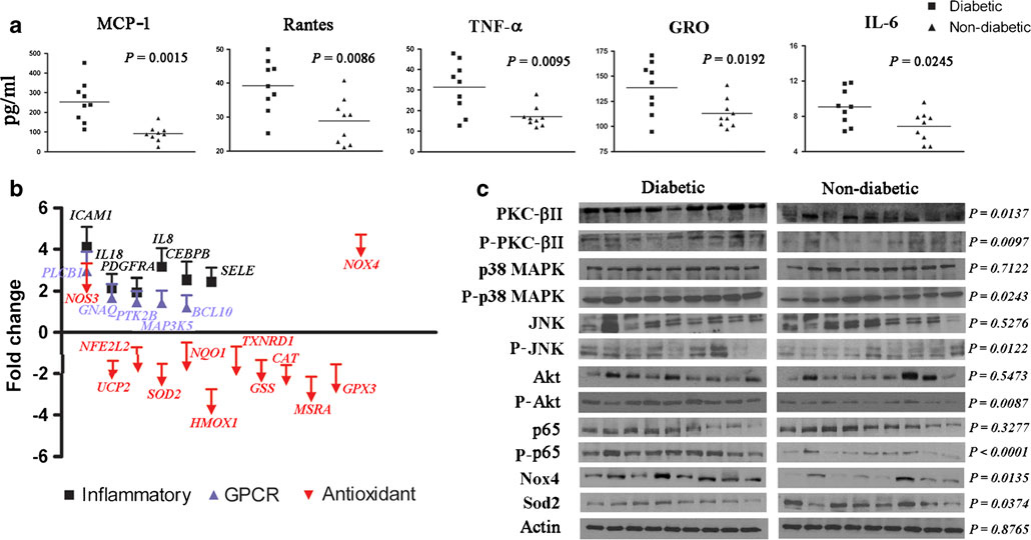
Diabetic kidneys with clinically normal renal function are inflamed. **a** Significantly higher concentrations of common proinflammatory cytokines were detected in fresh frozen kidneys of diabetic (*n* = 9) than in non-diabetic (*n* = 9) subjects using a custom Quantibody® Human Cytokine Array (RayBiotech, Inc.) **b** Transcript levels of genes implicated in inflammation (6 genes), G protein-coupled receptor (*GPCR*) signaling (5 genes), and antioxidant pathway (12 genes) were analyzed by qPCR array. Transcripts for genes implicated in inflammatory and GPCR pathways were upregulated in diabetic kidneys while transcripts encoding antioxidant enzymes were downregulated but Nox4, a superior generator, was upregulated. The fold change was calculated from expression levels in fresh frozen kidney tissues of six diabetic subjects (each positive for both HCMV and bacteria) compared with an equal number of non-diabetic subjects (negative for both HCMV and bacteria). **c** Kidney lysates and organelle-specific proteins of diabetic and non-diabetic subjects (*n* = 9 each) were assayed by western blot analysis with the indicated antibodies. All nine diabetic samples were positive for HCMV, bacteria or both. All nine non-diabetic samples were negative for both HCMV and bacteria. The *P* values compare the expression levels of diabetic and non-diabetic subjects and were calculated based on actin-normalized densitometry values of each group. Western blot experiments for each antibody were repeated at least thrice. Representative blots for each antibody are shown

Chronic inflammation is a hallmark of diabetes [25]. To investigate effects of inflammation on immunity in diabetes, we assayed adhesion molecules, cytokines, chemokines, and their receptors in renal tissues. Diabetic kidneys had significantly higher levels of proinflammatory molecules (MCP-1, Rantes, TNF-α, GRO, and IL-6) compared with controls (Fig. 2a). Transcript levels of *IL8*, *IL18*, *SELE*, *ICAM1*, *CEBPB*, and *PDGFRA* were all higher in diabetic kidneys (Fig. 2b).

### Inflammation in diabetic kidneys is associated with increased GPCR and MAP kinase activities, and oxidative stress

Signaling pathways associated with chronic inflammation were observed in diabetic kidneys, evidenced by increased protein levels and phosphorylation of PKC-βII (Fig. 2c) and NFκB (p65), increased expression of *PRKCA*, *PRKCB*, and *PRKCQ* (Fig. 1b) and increased phosphorylation of MAPK14 (p38 MAPK) and c-Jun N-terminal kinase (JNK). Concomitantly, GPCR signaling molecules that regulate these pathways were induced. *GNAQ* and *PLCB1* were upregulated, as were activators of NFκB (*BCL10* and *PTK2B* (*PYK2*)), p38 MAPK (*MAP3K5* or *ASK1*), and JNK (*PTK2B*) (Fig. 2b). Akt activation in diabetic kidneys was noteworthy because it is known to activate NFκB, JNK, and extracellular signal-regulated kinase (Fig. 2c).

Increased oxidative stress activating NFκB has been implicated in diabetes complications [26]. Diabetic kidneys had increased transcript and membrane-bound protein levels of Nox4, the renal-specific generator of cytosolic reactive oxygen species (ROS) and reduced expression of SOD2, *UCP2*, *GPX3*, *NQO1*, and *CAT* (Fig. 2b, c). Impaired anti-oxidative capacity was implied by decreased expression of *NFE2L2*, *HMOX1*, *GSS*, *MSRA*, and *TXNRD1* (Fig. 2b). In aggregate, these results point to increased oxidative stress in diabetic kidneys.

### The paradox of heightened inflammation and impaired immunity in diabetes

**Fig. 3:**
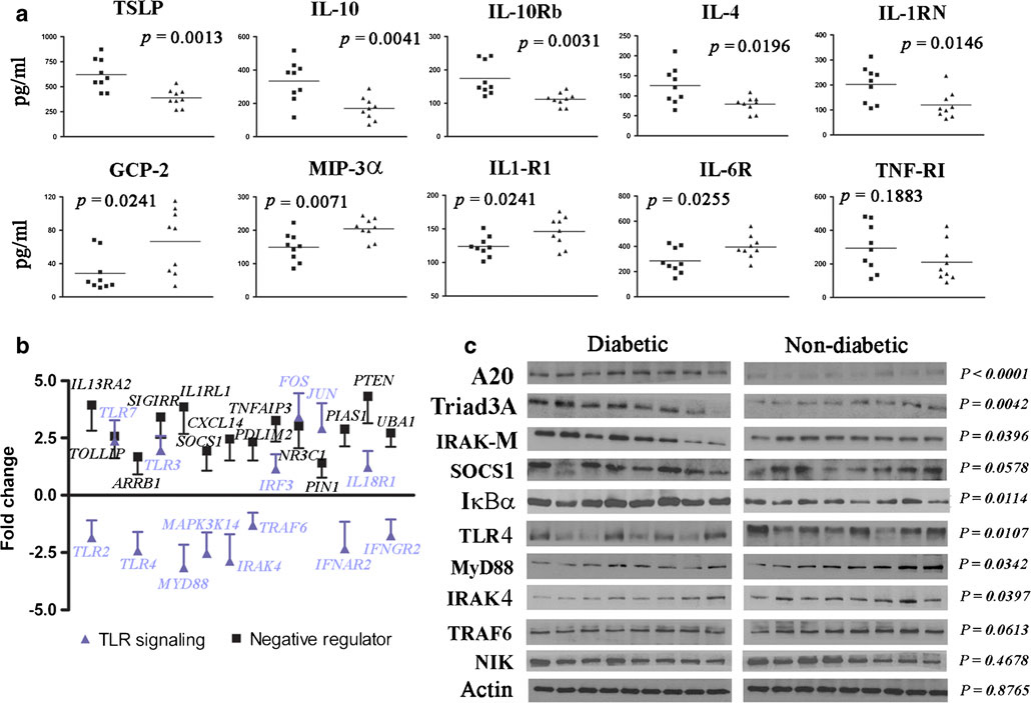
Upregulation of several negative regulators of inflammation is associated with impaired Toll-like receptor signaling in diabetic kidneys. **a** Significantly higher expression of immunomodulatory and anti-inflammatory cytokines and signaling molecules (TSLP, IL-10, IL-10Rb, IL-4, and IL-1RN) but suppressed expression of antimicrobial cytokines (GCP-2 and MIP-3α) and cytokine receptors (IL1-R1, IL-6R) in fresh frozen diabetic kidneys (*n* = 9 (*black squares*); *cf*. non-diabetic kidneys, *n* = 9 (*triangles*)) using a custom Quantibody® Human Cytokine Array (RayBiotech, Inc.). **b** Diabetic kidneys showed significant upregulation of all negative regulators tested (14 genes) but downregulation of 8 of 14 genes involved in TLR signaling. Fold change for each gene represents the average transcript levels of fresh frozen diabetic kidneys compared with non-diabetic kidneys (*n* = 6 each). Six diabetic subjects (each positive for both HCMV and bacteria) were compared with an equal number of non-diabetic subjects (negative for both HCMV and bacteria). **c** Western blot analysis of negative regulators of inflammation (A20, Triad3A, IRAK-M, SOCS1, and IκBα) and TLR signaling molecules (TLR4, MyD88, IRAK4, TRAF6, and NIK) confirmed their higher and lower expression, respectively, in fresh frozen diabetic kidneys. The *P* value for each paired comparison was calculated from actin-normalized densitometry values of diabetic (*n* = 8) and non-diabetic (*n* = 8) kidneys. All eight diabetic samples were positive for HCMV, bacteria or both. All eight non-diabetic samples were negative for both HCMV and bacteria. Western blot experiments for each antibody were repeated at least thrice. Representative blots for each antibody are shown

The foregoing changes in diabetic kidneys raise a paradox. How do heightened inflammatory responses in diabetes coexist with increased susceptibility to infections, since the former should, in theory, combat microbial invasions more effectively? We hypothesized that the dual effects of TGF-β overexpression could help to resolve this paradox (Fig. 1). TGF-β potentiates inflammation and induces renal fibrosis but is also important in regulating immune homeostasis [27]. Assaying other immunomodulatory molecules, we showed significant upregulation in diabetic kidneys of IL-4, TSLP, IL-1RN, IL-10, and IL-10Rb, which have anti-inflammatory and immunosuppressive actions (Fig. 3a). Conversely, IL1-R1 and IL6-R1 were downregulated in diabetic kidneys. Expression of TNFRI, the major TNF-α receptor, was not significantly altered. Antibacterial chemokines, GCP-2 (CXCL6), and MIP-3α (CCL20), were reduced in diabetic kidneys (Fig. 3a).

Next, we examined negative regulators of inflammatory signaling. In diabetic kidneys, suppression of Toll-like receptor (TLR) signaling was associated with overexpression of its negative regulators (Fig. 3b, c). The level of A20 was higher in diabetic kidneys and correlated with lower transcript and protein levels of TRAF6. Negative regulators of proinflammatory surface receptors were also overexpressed in diabetic kidneys. Triad3A (RNF216) was significantly higher in diabetes and may have contributed to reduced levels of TLR4 protein (Fig. 3c). Similarly, IRAK-M was overexpressed while IRAK4 protein was repressed in diabetic kidneys. Upregulation of transcripts of *IL1RL1* (*ST2L*) and *SIGIRR* of the TIR superfamily, may also modulate TLR signaling in diabetic tissues (Fig. 3b). Upregulation of *IL1RL1* and *SIGIRR* was consistent with lower protein levels of MyD88, TLR4, IRAK4 and TRAF6 in diabetic kidneys (Fig. 3c). Overexpression of *TOLLIP* in diabetes was yet another possible mechanism for inhibition of TLR2- and TLR4-mediated NFκB activation.

SOCS1, a negative regulator of several inflammatory pathways, was more highly expressed in diabetic kidneys (Fig. 3b, c). PTEN transcription was increased. Other known negative regulators of proinflammatory pathways upregulated in diabetic kidneys included *ARRB1*, *PIN1* and *UBA1* (Fig. 3b). Taken together, our results showed that multiple counter-regulatory mechanisms against heightened inflammation were excessively activated in diabetic kidneys, causing loss of immunological homeostasis that could impair immune defenses.

### Increased expression of negative regulators is associated with impaired immunity and cryptic microbial infections in diabetic kidneys

**Fig. 4:**
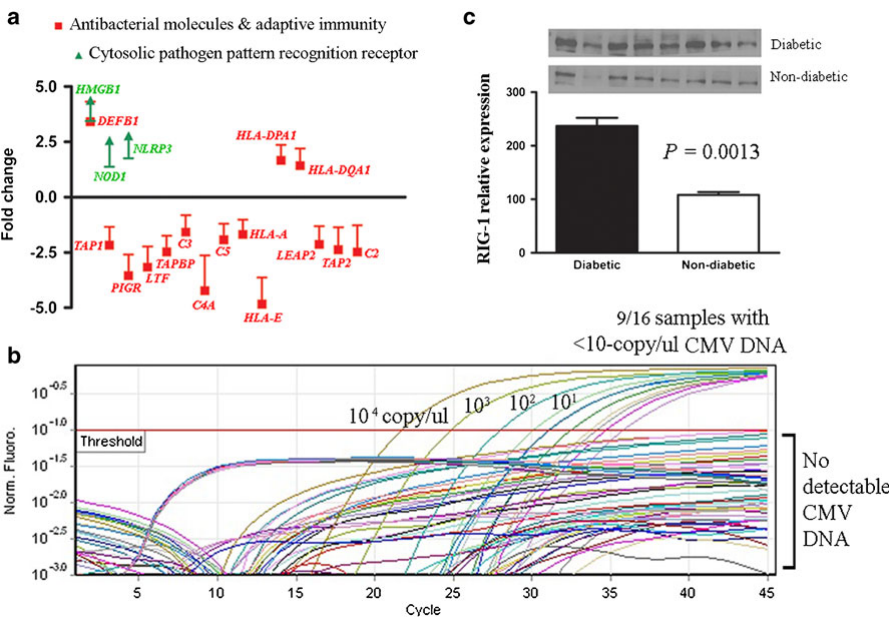
Immune-impaired diabetic kidneys had cryptic microbial infections. **a** Transcript levels of genes encoding antimicrobial peptides, serum complement factors, antigen presentation proteins (15 genes), and cytosolic pathogen pattern recognition receptors (3 genes) were assayed on fresh frozen kidney tissues with qPCR array. The fold change was calculated from renal expression levels of six diabetic subjects (each positive for both HCMV and bacteria) compared with an equal number of non-diabetic subjects (each negative for both HCMV and bacteria). **b** A clinical diagnostic kit, artus® CMV RG PCR kit (Qiagen GmbH), was used to detect HCMV DNA in 16 diabetic (nine fresh frozen and seven FFPE) and 3 non-diabetic kidneys (all fresh frozen). The diagnostic kit detects specific amplification of a 105-bp DNA region of the HCMV genome. Real-time PCR was performed on Rotor-Gene™ 3000 (Roche) as instructed. Data were analyzed with Rotor-Gene™ software. **c** The relative expression levels of RIG-1 were derived from the average of actin-normalized densitometry values of diabetic and non-diabetic kidneys (*n* = 8 each). All eight diabetic samples were positive for HCMV, bacteria or both. All eight non-diabetic samples were negative for both HCMV and bacteria. Western blot analysis was performed at least thrice. A representative blot is shown

**Fig. 5:**
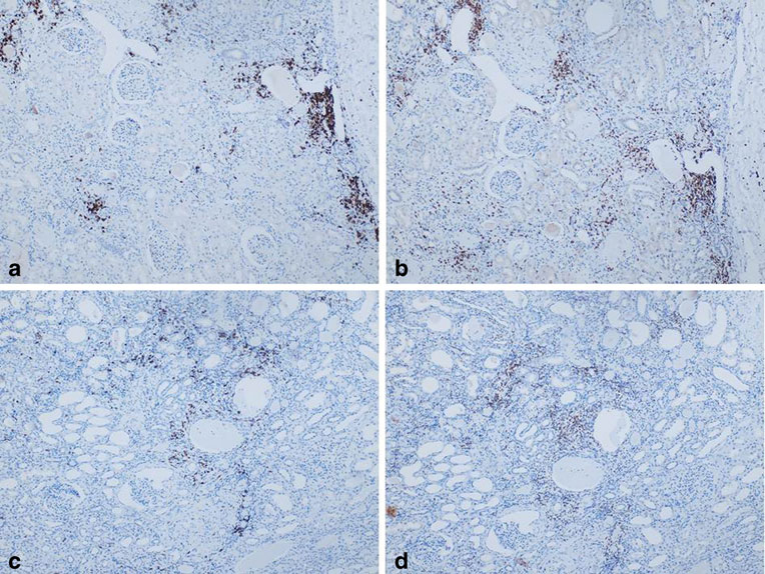
T and B lymphocyte infiltrations of diabetic kidneys. Immunohistochemical staining for B (**a**, **c**) and T lymphocytes (**b**, **d**) of two diabetic kidneys (*top* case 05-PB20861 and *bottom* case 06-PB627) with evidence of bacterial and HCMV DNA. Primary antibodies were monoclonal anti-CD20 (B lymphocytes; Dako M0755, clone L26; 1:200 dilution) and polyclonal anti-CD3 (T lymphocytes; Dako A0452; dilution 1:200). The reporter system was EnVision™ (Dako; horseradish peroxidase and 3,3′diaminobenzidinetetrahydrochloride). Counterstain was hematoxylin. Original magnification was ×100

Transcription of genes encoding antimicrobial peptides (*LEAP2, PIGR*, and *LTF*) and complement factors (*C2*, *C3*, *C4A*, and *C5*), were all repressed in diabetic kidneys, except for *DEFB1*, which is known to be glucose-induced (Fig. 4a). Adaptive immunity was also impaired. Genes encoding antigen-presenting proteins, *TAP1*, *TAP2*, *TAPBP*, *HLA-A*, and *HLA-E*, were repressed in diabetic kidneys. High expression of HLA-DPA1 and HLA-DQA1 in diabetic kidneys suggested internalized microorganisms in renal cells. To investigate clinically silent infections in diabetic kidneys, assay for HCMV DNA using a clinical diagnostic kit revealed the cryptic presence of HCMV DNA in six of nine fresh frozen and three of seven FFPE samples, giving a total positivity of 9 of 16 (56 %) in diabetic kidneys (Fig. 4b). Quantitative PCR using bacteria-specific primers [23] showed that 13 of 19 (68 %) of diabetic kidneys and 2 of 11 (18 %) of non-diabetic kidneys had >50 pg of bacterial DNA/50 ng total DNA. In contrast, 9 of 11 (82 %) of non-diabetic kidneys and 5 of 5 negative controls (lymphocyte DNA from unrelated non-diabetic subjects) had <1 pg of bacterial DNA/50 ng total DNA. Among diabetic kidneys tested for both HCMV and bacteria (*n* = 16), six had both HCMV and bacterial DNA, three had HCMV DNA only, and seven had bacterial DNA only. Consistent with the presence of cryptic infections, cytoplasmic pathogen-associated molecular pattern (PAMP) receptors (RIG-1, *TLR3*, *TLR7*, *HMGB1*, *NOD1*, and *NLRP3*) were upregulated in diabetic kidneys positive for HCMV and/or bacterial DNA (Fig. 3b, and 4a, c). Figure 5 shows T and B lymphocyte infiltration in two diabetic kidneys that were positive for HCMV and bacterial DNA.

### PKC inhibition and antioxidant reverse impaired immune responses in renal epithelial cells

**Fig. 6:**
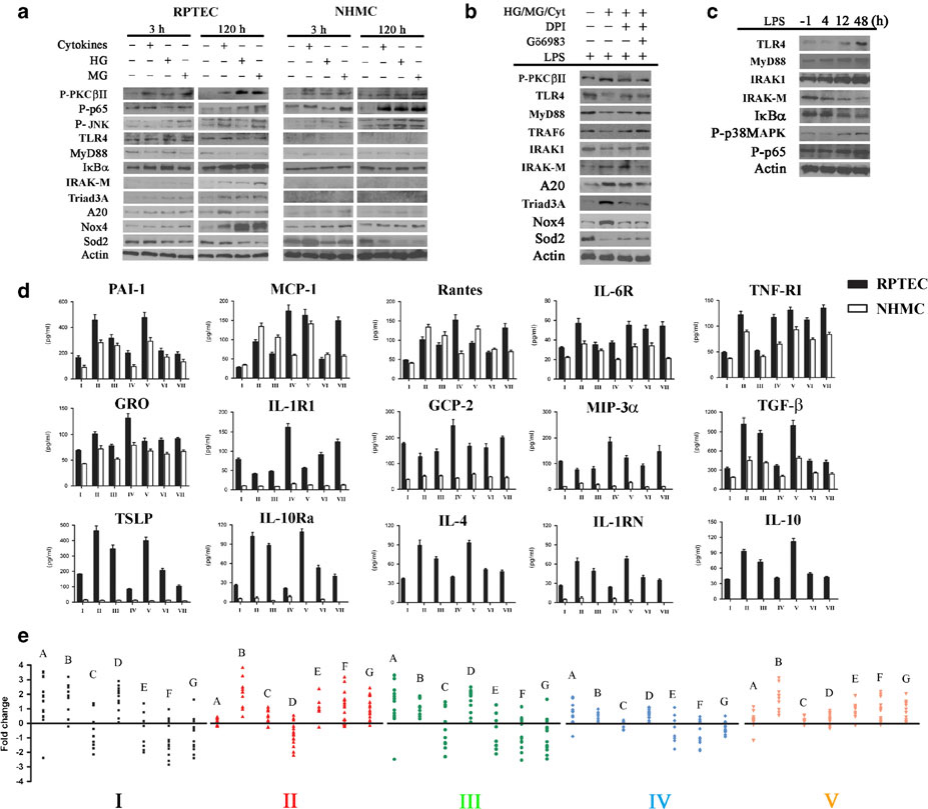
Renal tubular epithelial cells, but not mesangial cells, modulate overt inflammation via negative feedback mechanisms that impair immunity. **a** Human renal primary tubular epithelial cells (*RPTEC*) and mesangial cells (*NHMC*; Lonza) were cultured with diabetes-relevant concentrations of cytokines (TNF-α, 50 pg/ml; IL-6, 20 pg/ml; and IL-1β, 20 pg/ml), high glucose (*HG*; 20 mM) or methylglyoxal (*MG*; 10 μM) for 3 h or 5 days (120 h). Protein lysates from each condition (*n* = 4) were prepared for western blot analysis using the indicated antibodies (*n* ≥ 3 for each antibody). **b** RPTEC were first exposed to a combination of cytokines, high glucose, and methylglyoxal at concentrations stated in (**a**) for 5 days. The cells were further cultured in fresh medium in the presence of either diphenyleneiodonium chloride (*DPI*; 10 μM) or combined with Gö6983 (10 nM) for 1 day before challenge with lipopolysaccharide (*LPS*; 1 μg/ml) for 2 h. Protein lysates (*n* = 4 for each condition) were analyzed by western blotting (≥3 immunoblots for each antibody). **c** RPTEC cultured in the combined presence of cytokines, HG and MG at the aforementioned concentrations for 5 days were challenged with a higher dose of LPS (10 μg/ml) for the indicated time points (*n* = 4 for each time point). Minus 1 h denotes cells that were not exposed to LPS but were lysed for protein isolation one hour before similarly cultured cells were challenged with LPS. Protein lysates from each condition were prepared for western blotting as described (Figs. 2 and 3 legends). **d** RPTEC and NHMC were treated as follows before harvesting for protein and total RNA isolations (*n* = 4 for each condition). *I* Untreated control cells; *II* incubated with cytokines (TNF-α, 50 pg/ml; IL-6, 20 pg/ml; and IL-1β, 20 pg/ml), HG (20 mM), and MG (10 μM) for 5 days; *III* incubated with MG (10 μM) only for 5 days; *IV* challenged with LPS (10 μg/ml), lipoteichoic acid (*LTA*; 10 μg/ml), and poly(I:C) (10 μg/ml) for 2 h; *V* treated as in (*II*) before returning to normal culture medium for 1 day and subsequently challenged with microbial stimulants as in (*IV*); *VI* cultured as in (*II*) before treatment with DPI (10 μM) and Gö6983 (10 nM) for 1 day in fresh culture medium; *VII* cultured as in (*VI*) before challenge as in (*IV*). Protein lysates were assayed using quantitative protein arrays. **e** Results of qPCR array plotted as fold change (RPTEC of each condition *cf.* untreated RPTEC). Genes with similar functions or involvement in the same biological process were grouped. *A* Diabetes related, *B* inflammatory pathways, *C* antioxidant system, *D* negative immune regulators, *E* interleukin and cytokine receptors, *F* TLR signaling, and *G* antimicrobial molecules and adaptive immunity. Roman numerals (*I–V*) indicate the type of treatment. *I* RPTEC incubated with cytokines (TNF-α, 50 pg/ml; IL-6, 20 pg/ml; and IL-1β, 20 pg/ml), HG (20 mM), and MG (10 μM) for 5 days; *II* RPTEC challenged with LPS (10 μg/ml), LTA (10 μg/ml) and poly I/C (10 μg/ml) for 2 h; *III* RPTEC treated as in (*I*), returned to normal culture medium for 1 day and then challenged with microbial analogs as in (*II*); *IV* RPTEC cultured as in (*I*) before combined treatment with DPI (10 μM) and Gö6983 (10 nM) for 1 day in fresh culture medium; *V* RPTEC cultured as in (*IV*) before being challenged as in (*II*). The qPCR array experiment for each of the conditions was performed twice

To identify the resident cell type that plays a major role in the immune responses of whole kidney, we investigated human primary RPTEC and NHMC. RPTEC responded to diabetes-relevant concentrations of metabolic insults (high glucose (HG; 20 mM), methylglyoxal (MG; 10 μM), and cytokines (TNF-α, IL-6, and IL-1β)) not only by activating proinflammatory signaling molecules and increasing expression of proinflammatory cytokines but also by upregulating immunoregulatory molecules (e.g., IL-4, IL-10, TGF-β, and TSLP) and negative regulators of inflammation (e.g., IκBα, IRAK-M, Triad3A, and A20) (Fig. 6). These responses were significant after a 5-day stimulation with any single agent (Fig. 6a, e). In contrast, although mesangial cells exposed to the same agents showed proinflammatory responses, they expressed very low levels of negative regulators and immunoregulatory proteins (Fig. 6a, d). Comparing HG, MG, and cytokines, induction of proinflammatory genes and oxidative stress in both cell types was highest after a 5-day treatment with MG, followed by HG and cytokines (Fig. 6a). RPTEC had highest expression of negative regulators and lowest expression of TLR signaling molecules in response to MG. However, no significant change was observed in MG-treated NHMC. To determine if the immune response of RPTEC was impaired in conditions that simulated the metabolic milieu of diabetes, we compared protein and mRNA expression profiles of RPTEC challenged with LPS alone (Fig. 6b, c) or a combination of LPS, lipoteichoic acid, and poly(I:C) (Fig. 6d, e), before and after a 5-day incubation with HG, MG, and cytokines. In the same experiment, we also investigated the effects of DPI, an antioxidant that targets NADPH oxidase, alone or in combination with a PKC inhibitor, Gö6983, on the immune response of RPTEC. RPTEC exposed to conditions mimicking the diabetic metabolic milieu showed a delayed response to LPS stimulation. Activation of TLR leading to NFκB and p38 MAPK activation was evident only 12 h after LPS stimulation (Fig. 6c) compared with control-unexposed RPTEC that responded to LPS stimulation within 2 h (Fig. 6b). This delayed response of RPTEC in a diabetic milieu was associated with expression of negative regulators (Fig. 6c) and less robust expression of molecules associated with antimicrobial response (e.g., PIGR, LEAP2, LTF, C2, C3, C4A, C5, TAP1, TAP2, and TAPBP) when challenged with microbial analogs. This was associated with upregulation of multiple immunomodulatory cytokines, negative regulators of inflammation, increased ROS generation, and evidence of impaired intrinsic antioxidant capacity (Fig. 6d, e). The combined presence of Gö6983 and DPI (Fig. 6b, d, e), but not either agent alone (Fig. 6b), mitigated the proinflammatory response, reduced the expression of negative regulators, and restored the prompt immune response of RPTEC challenged with microbial stimulants. Collectively, these in vitro results point to inappropriately heightened counter-regulatory responses to inflammation as a cause of impaired renal epithelial immunity in diabetes and demonstrate reversal by pharmacological interventions directed at PKC activation and ROS generation.

## Discussion

Our results showed that kidneys of diabetic subjects overexpressed inflammatory markers, and had dysregulated signaling pathways involving GPCR, PKC-β, MAPKs (p38 MAPK and JNK), Akt, TLRs, and NFκB. Our data additionally revealed Nox4-induced ROS generation, hyperglycemia-induced mitochondrial dysfunction leading to reduced antioxidant capacity, NFAT5 overexpression, and glucose-induced upregulation of EGR1. EGR1 heterodimerizes with NFAT5 to induce proinflammatory cytokine gene expression [28].

Our results help to explain the association of impaired immunity with frequent infections by showing that inappropriate induction of immunomodulatory molecules and negative regulators of inflammatory pathways accompany heightened inflammatory responses in diabetic kidneys. This suggests that clinically subtle immunocompromise can result from conserved regulatory responses evolved for combating biological stressors, e.g., hyperglycemia and chronic inflammation. Indeed, immune responses and metabolic regulation are interdependent in maintaining homeostatic equilibrium [25]. Alternative mechanisms of impaired immunity in diabetes are known. Hyperglycemia induces overexpression of endogenous TLR4 ligands, fibronectin, and biglycan. Chronic TLR4 activation leads to hypo-responsiveness to PAMPs [29, 30]. This suggests an explanation for our results showing downregulation of surface TLRs (e.g., TLR2 and TLR4) in diabetic kidneys. In contrast, the expression of cytosolic, endosome-bound TLRs, e.g., TLR3 and TLR7, and an intracellular RNA helicase, RIG-1, were induced in infected diabetic kidneys because they recognize intracellular microbial patterns [31].

Recognizing the inherent heterogeneity of whole tissue analysis, we complemented whole kidney studies with in vitro experiments on subtypes of primary human renal cells. Our results confirmed the active role of renal epithelial cells as key players in renal tissue immunity, in contrast to renal mesangial cells having a more passive function. Although mesangial cells are metabolically vulnerable because they cannot restrain intracellular glucose transport and hence tend to overexpress markers of inflammation [32], our data show that their role in tissue immunity is minimal. We propose that renal epithelial cells respond to heightened inflammation and oxidative stress by upregulating immunomodulatory molecules that may influence immune cells of the microenvironment to favor T_H_2 responses. Concurrently, epithelial cells protect themselves from excessive inflammation by expressing a myriad of negative regulators to inhibit inflammatory signaling, particularly the TLR pathway [33]. While it may be effective in helping to extinguish inflammation, this strategy is not without cost to the immune system and the diabetic host, as our findings of cryptic HCMV and/or bacterial infections show. As about one third of diabetic patients develop nephropathy and end-stage renal failure, it is possible that impaired immunity and cryptic infections are contributory factors.

Our in vitro experiments confirmed the role of inflammation and oxidative stress as the main instigators of impaired immunity in diabetes as combined pharmacological intervention with a PKC inhibitor and an antioxidant targeting the ROS generator, NADPH oxidase, but not either agent alone, reversed the molecular dysregulation initiated by common molecular assaults of diabetes and restored a prompt immune response of renal epithelial cells. Our data further confirm and highlight the role of MG in impairing immune functions in diabetes [34]. We show MG to be potent in triggering overexpression of inflammatory molecules and ROS in renal epithelial and mesangial cells, with subsequent impairment of TLR signaling in epithelial cells.

**Fig. 7:**
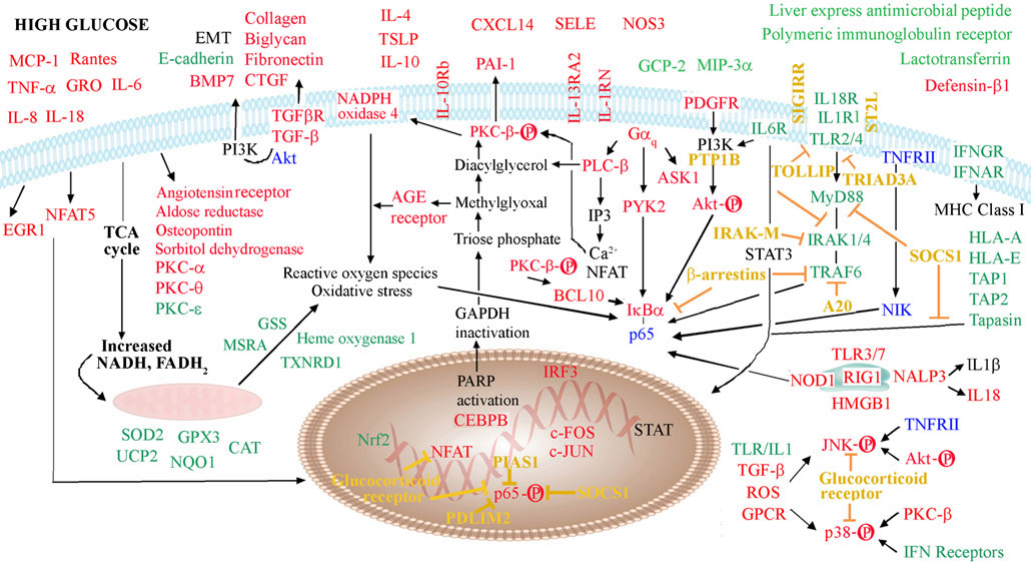
Proposed model showing molecular mechanisms of dysregulated immune pathways in diabetic renal tissues. Hyperglycemia alters four molecular processes implicated in the pathogenesis of diabetes complications: (1) polyol pathway, (2) increased expression of the receptor for advanced glycation end-product, (3) hexosamine pathway, and (4) protein kinase C (*PKC*) signaling. This results in chronic inflammation, accumulation of extracellular matrix proteins, tissue remodeling, mitochondrial dysfunction, and oxidative stress. Chronic activation of the key switches of proinflammatory responses, e.g., PKC-β, NF-κB, MAPKs, AKT, G protein-coupled receptors (*GPCRs*), and Toll-like receptors (*TLRs*) are induced. Oxidative stress from increased generation of reactive oxygen species (*ROS*), impaired mitochondrial function and diminished antioxidant capacity further exacerbates the proinflammatory state, especially when ROS and PKC-β are mutually activating and oxidative stress activates NF-κB. To combat and protect resident cells and tissue from unresolved inflammation, anti-inflammatory mechanisms involving negative regulators of proinflammatory molecules and signaling pathways (*yellow letters*), immunomodulatory molecules (e.g., IL4, TSLP, and CXCL14), and decoy receptors (e.g., IL1-R1, IL6-R1, and IL13RA2) are chronically activated. These inherent protective mechanisms however compromise antimicrobial defense by interfering with TLR and NF-κB signaling that in turn reduce expression of key players of innate and adaptive immunity (e.g., circulating antimicrobial peptides and complement factors, and proteins with important roles in antigen presentation). Impaired immunity predisposes to infections that activate cytoplasmic pathogen-associated molecular pattern receptors which recognize internalized bacterial and viral molecules. Information was derived from Ingenuity Pathways Knowledge Base and published literature. *Red letters* denote upregulation; *green letters*, downregulation; *black letters*, mechanisms/modulators not analyzed in this study

In conclusion, our data suggest an integrated model for diabetes-impaired immunity (Fig. 7). Hyperglycemia induces changes in multiple intrarenal pathways involving mechanisms implicated in tissue damage [32]. These result in mitochondrial dysfunction and oxidative stress, a net effect of diminished antioxidant capability and accumulation of intracellular ROS. Other crucial effects are accumulation of extracellular matrix proteins and proinflammatory responses involving activation of NF-κB, MAPKs, and Akt. Activation of GPCR signaling by ligands such as cytokines, chemokines, lipid and growth factors, and oxidative stress create a vicious cycle of chronic inflammation. In turn, renal epithelial cells mount protective mechanisms to combat these stressors by stimulating the capacity to restore immune homeostasis through inducing T_H_2 and anti-inflammatory responses, and overexpressing negative regulators that intercept proinflammatory responses, especially those directed at TLR signaling and NF-κB. These may interfere with selective transcription of genes regulated by NFκB, other inflammatory transcription factors and the physiological synergy among them [35]. Thus, the expression of target genes of innate and adaptive immunity could be compromised, resulting in increased risk of infections. Internalized bacterial and viral molecules during the course of renal infection may activate cytosolic PAMP receptors, triggering yet another wave of inflammatory response.

## Electronic supplementary material

Below is the link to the electronic supplementary material.

ESM 1 (DOCX 106 KB)

Supplementary Fig. 1 (GIF 1.87 MB)

High resolution image file (TIF 1.23 MB)
