# Supplementary material for: Negative regulatory responses to metabolically triggered inflammation impair renal epithelial immunity in diabetes mellitus
Source: J Mol Med (Berl). 2012 Nov 14;91(5):587–98. doi: 10.1007/s00109-012-0969-x (PMC3644409; doi:10.1007/s00109-012-0969-x)
Supplement: Supplementary file 1 — (DOCX 106 KB) [file 109_2012_969_MOESM1_ESM.docx]

**Supplemental Information**

**Table S1.** Clinical profiles of study subjects and analyses performed on fresh frozen and formalin-fixed paraffin-embedded tissues

Legend:

C=Chinese; M=Malay; I=Indian; E=Eurasian; Pos=Positive; Neg=Negative; Fr=Fresh frozen; FFPE=Formalin-fixed paraffin-embedded; NT=Not tested.

**Table S2.** List of genes and proteins analyzed

| **Gene/Protein** | **Name, Function(s)* and brief description** |
| --- | --- |
|  | **Diabetes-associated** |
| AKR1B1 | *aldo-keto reductase family 1, member B1 (aldose reductase)*  Catalyzes NADPH-dependent reduction of carbonyl compounds, including the reduction of glucose to sorbitol  Over-expressed in diabetes (Brownlee) |
| SORD | *sorbitol dehydrogenase*  Converts sorbitol to fructose; part of the polyol pathway  Over-expressed in diabetes (Brownlee) |
| AGER | *advanced glycosylation end product-specific receptor*  Modulates interactions of advanced glycation end products (AGE)  Over-expressed in diabetes (Brownlee) |
| TGFB1 | *transforming growth factor, beta 1*  Multifunctional protein that controls proliferation and differentiation in many cell types  Intracellular glycosylation by OGT increases donation of GlcNAc moieties to transcription factor such as SP1, which then increases expression of PAI-1 and TGFb1.  Upregulated in human diabetic nephropathy (Lindenmeyer MT (Kretzler M), J Am Soc Nephrol 18: 1765-1776, 2007).  Induces basement membrane thickening (increased ECM deposition), fibrin deposition, tissue fibrosis and glomerular sclerosis |
| TGFBR1 | *transforming growth factor, beta receptor 1*  Transduces the TGFB1, TGFB2 and TGFB3 signal from the cell surface to the cytoplasm and is thus regulating a plethora of physiological and pathological processes including cell cycle arrest in epithelial and hematopoietic cells, control of mesenchymal cell proliferation and differentiation, wound healing, extracellular matrix production, immunosuppression and carcinogenesis.  Regulates epithelial to mesenchymal transition  See TGFB1 |
| PAI1/ SERPINE1 | *serpin peptidase inhibitor, clade E (nexin, plasminogen activator inhibitor type 1), member 1*  Serine proteinase inhibitor; a major inhibitor of fibrinolysis  Over-expressed in diabetes |
| PRKCA | *protein kinase C, alpha*  Serine/threonine protein kinase; activated by calcium, phospholipid and diacylglycerol; involved in cell proliferation, apoptosis, differentiation cell adhesion and inflammation |
| PRKCB | *protein kinase C, beta*  Serine/threonine protein kinase; activated by calcium, phospholipid and diacylglycerol; involved in regulating B-cell receptor signalosome and oxidative stress-induced apoptosis  High glucose concentration leads to *de novo* diacylglycerol synthesis, which activates protein kinase C. PKC activation (mainly β- and δ isoforms) resulted in c-fos and c-jun expressions, which combine in homo/heterodimers to form AP-1 transcription complex. This complex binds to, and induces expression of TGFβ, fibronectin, laminin and ECM matrix proteins. Several other markers for diabetes are also induced: endothelin 1 (see Sasser JM, Endothelin A Receptor Blockade Reduces Diabetic Renal Injury via an Anti-Inflammatory Mechanism. JASN 2007, and Chen S, Differential activation of NF-kB and AP-1 in increased fibronectin synthesis in target organs of diabetic complications. Am J Physiol Endocrinol Metab 2003: dependent on ET-mediated receptor signaling), VEGF, Serpine 1, reduced fibrinolysis, activation of NF-kB and NADPH oxidases (increased ROS). Expression of endothelial nitric oxide synthetase (eNOS) is repressed. eNOS and prostacyclin synthase are 2 important antiatherogenic enzymes that prevent vascular pathology. Therefore, PKCβ activation leads to oxidative stress, expression of inflammatory mediators, cellular proliferation and tissue fibrosis (via upregulation of TGFβ). |
| PRKCE | *protein kinase C, epsilon*  Calcium-independent, phospholipid- and diacylglycerol-dependent serine/threonine-protein kinase that plays essential roles in the regulation of multiple cellular processes linked to cytoskeletal proteins; involved in immune response, cancer cell invasion and regulation of apoptosis.  Downstream of TLR4, plays an important role in the lipopolysaccharide (LPS)-induced immune response by phosphorylating and activating TICAM2/TRAM, which in turn activates the transcription factor IRF3 and subsequent cytokines production.  See PRKCB |
| PRKCQ | *protein kinase C, theta*  Serine/threonine protein kinase; activated by calcium, phospholipid and diacylglycerol; mediates non-redundant functions in T-cell receptor signaling; activates NF-kappa-B  See PRKCB |
| EDN1 | *endothelin 1*  A secreted peptide and potent vasoconstrictor produced by vascular endothelial cells  See PRKCB |
| VEGFA | *vascular endothelial growth factor A*  Growth factor; promotes angiogenesis, vasculogenesis, endothelial cell proliferation, permeabilization of blood vessels and cell migration  See PRKCB |
| SPP1 | *secreted phosphoprotein 1 (osteopontin)*  Cytokine; enhances production of IFN-gamma and IL-12; reduces IL-10 production |
| EGR1 | *early growth response 1*  Regulates transcription of genes involved in mitogenesis and differentiation  Hasan RN, Phukan S, Harada S. Differential regulation of early growth response gene-1 expression by insulin and glucose in vascular endothelial cells. Arterioscler Thromb Vasc Biol 2003; 23: 988–993.  Decker EL et al. Early growth response proteins (EGR) and nuclear factors of activated T cells (NFAT) form heterodimers and regulate proinflammatory cytokine gene expression. Nucleic Acid Res 2003  Khachigian LM, Egr-1-induced endothelial gene expression: A common theme in vascular injury. Science 1996: Egr-1 up-regulated expression of PDGF-B and other potent mediators in mechanically injured arterial endothelial cells. |
| NFAT5 | *nuclear factor of activated T-cells 5, tonicity-responsive*  Transcription factor; regulates gene expression induced by osmotic stress in mammalian cells  Induced by hyperosmosis and not calcium signaling  Lopez-Rodriguez, Bridging the NFAT & NF-kB Families-NFAT5 Regulates Cytokine Transcriptionn in Response to Osmotic Stress. Immunity 2001.  NFAT5 levels are increased in osmotically-stressed cells. A variety of osmotically-regulated genes (e.g. aldose reductase) contains NFAT5 binding elements.  Macian F, NFAT proteins: key regulators of T-cell development and function. Nat Rev Immunol 2005; 5:472-484) |
| AGTR1 | *Angiotensin II receptor, type 1*  Involved in G-protein signaling, coupled to IP3 second messenger (PLC) |
| INSR | *Insulin receptor*  Receptor tyrosine kinase; mediates pleiotropic actions of insulin  Down-regulation is implicated in insulin resistance and impaired insulin signaling |
| BGN | *Biglycan*  Involved in collagen assembly  Biglycan is a small leucine-rich proteoglycan, a ubiquitous ECM component.  Schaefer L, The matrix component biglycan is proinflammatory & signals through TLR 2 & 4 in macrophages. JCI 2005: evidence that matrix component can be a ligand for TLR. |
| FN1 | *fibronectin 1*  Binds cell surfaces and collagen; involved in cell adhesion and motility  See PRKCB |
| COL1A1 | *collagen, type I, alpha 1*  A fibril-forming collagen found in most connective tissues; a component of basement membrane  CEBPB stimulates expression of this gene by binding to the promoter and upstream element (see CEBPB) |
| THBS1 | *thrombospondin 1*  Adhesive glycoprotein; mediates cell-cell and cell-matrix interactions  Integrin ligand |
| E-cadherin | *cadherin 1, type 1, E-cadherin (epithelial)*  Cadherins are calcium-dependent cell adhesion proteins. Involved in mechanisms regulating cell-cell adhesions, mobility and proliferation of epithelial cells. Has a potent invasive suppressor role. |
| BMP-7 | *bone morphogenetic protein 7*  Induces cartilage and bone formation. May be the osteoinductive factor responsible for the phenomenon of epithelial osteogenesis. Plays a role in calcium regulation and bone homeostasis  Bone morphogenetic proteins (BMPs) are pleiotropic secreted proteins, structurally related to transforming growth factor (TGF)-beta and activins. Influence inflammatory processes in adults due to their chemotactic activity on fibroblasts, myocytes, and inflammatory cells.  Functions to reduce macrophage infiltration and tissue damage in animal models of renal failure |
|  | **Inflammation** |
| ICAM1 | *intercellular adhesion molecule 1*  Cell surface glycoprotein expressed by a variety of hematopoietic and nonhematopoietic cells, including B and T cells, dendritic cells, macrophages, fibroblasts, keratinocytes and endothelial cells. |
| IL-18 | *Interleukin 18*  Augments natural killer cell activity; stimulates IFN-γ production  IL18 is structurally related to IL1 but unlike IL1, its major biologic functions include enhancing IFNγ production by T cells and promoting differentiation of IFNγ-producing TH1 CD4+ T cells. |
| IL-8 | *Interleukin 8*  Chemotactic factor for neutrophils, basophils and T cells  Kooten C, Cytokine cross talk between tubular epithelial cells and interstitial immunocompetent cells. Curr Opin Nephrol Hepertens 2001, Figs 1 & 2. |
| IL-6 | *Interleukin 6*  Cytokine with pleiotropic actions; differentiates B cells; induces acute phase response |
| SELE | *Selectin E*  Cell surface glycoprotein; mediates adhesion of neutrophils to endothelium  Selectin E is expressed by endothelium activated by cytokines such as TNF and IL1 (Leukocyte migration through endothelium (see Abbas, pg 31) |
| PDGFRA | *platelet-derived growth factor receptor, alpha polypeptide*  Receptor tyrosine kinase; cell surface receptor for PDGFA; role in chemotaxis, cell proliferation and migration  Studies in knockout mice, where homozygosity is lethal, indicate that the alpha form of the platelet-derived growth factor receptor is particularly important for kidney development. |
| CEBPB | *CCAAT/enhancer binding protein (C/EBP), beta*  Transcriptional activator of genes involved in inflammation and immunity  See COL1A1 |
| MCP-1 | *chemokine (C-C motif) ligand 2 (CCL2)*  Chemotactic factor for monocytes and basophils  Expression regulated by NFκB. |
| CCL5/  Rantes | *chemokine (C-C motif) ligand 5 (CCL5)*  Chemoattractant for monocytes, memory T-helper cells and eosinophils  Upregulated in human diabetic nephropathy |
| GRO | *chemokine (C-X-C motif) ligand 1*  Growth factor; chemoattractant for neutrophils; involved in inflammation |
| TNF-α | *Tumor necrosis factor, alpha*  Proinflammatory cytokine; mainly secreted by macrophages; involved in inflammatory and immune responses  Expression regulated by NFκB. |
| RELA/p65 | *v-rel reticuloendotheliosis viral oncogene homolog A (avian); Nuclear factor NF-kappa-B p65 subunit*  Pleiotropic transcription factor involved in inflammation, immunity and many other biological processes  NFkB activation is anti-apoptotic and pro-inflammatory. In the face of chronic infection, NFkB activation may be viewed as a protective mechanism in the course of diabetes (see Bierhaus A, Diabetes-associated sustained activation of the transcription factor nuclear factor-kB. Diabetes 2001, Discussion) |
|  | **G protein-coupled receptor (GPCR) signaling** |
| PKC-βII | *protein kinase C, beta*  Serine/threonine protein kinase; activated by calcium and diacylglycerol; involved in many processes including B cell activation  Huang HC, Regulation of the antioxidant response element by protein kinase C-mediated phosphorylation of NF-E2-related factor 2. PNAS 97: 12475-80, 2000: PKC-directed phosphorylation of Nrf2 may be a critical event for the nuclear translocation of this transcription factor in response to oxidative stress. (see also NFE2L2)  PKCβ activation leads to oxidative stress, expression of inflammatory mediators, cellular proliferation and tissue fibrosis (via upregulation of TGFβ) |
| P38MAPK/ MAPK11 | *Mitogen-activated protein kinase p38 beta / mitogen-activated protein kinase 11*  Serine/threonine protein kinase; essential component of MAP kinase signal transduction pathway; important role in response to proinflammatory cytokines  Activates transcription factors such as CREB1, ATF1, the NF-kappa-B isoform RELA/NFKB3, STAT1 and STAT3  The p38 MAPKs are activated by lipopolysaccharide |
| JNK/  MAPK8 | *JUN N-terminal kinase / mitogen-activated protein kinase 8*  Serine/threonine protein kinase; stimulated by proinflammatory cytokines; involved in multiple processes e.g. cell proliferation, migration and differentiation  JNK 1,2 and 3 (sometimes known as SAPKs or stress-activated kinases) and the p38  MAPKs (α-, β-, δ-, γ-isoforms) are activated by UV irradiation, inflammatory cytokines and hyperosmolarity. |
| AKT1/  PRKBA | *v-akt murine thymoma viral oncogene homolog 1 / Protein kinase B alpha*  Serine/threonine kinase; activated by phosphatidylinositol-3-kinase; regulates many processes e.g. cell proliferation and survival, metabolism, growth and angiogenesis via phosphorylation of >100 substrates. |
| PLCB1 | *phospholipase C, beta 1 (phosphoinositide-specific)*  Catalyzes the formation of two second messengers, inositol 1,4,5-trisphosphate and diacylglycerol, from phosphatidylinsolitol 4,5-bisphosphate  Involves in prostaglandin synthesis  See Marinissen MJ, G-protein-coupled receptors and signaling networks-emerging paradigms. Trends Pharmacol Sci 2001, Fig. 1 & 2.  Hydrolyzes the phosphatidylinositol 4,5-bisphosphate (PIP2) to generate 2 second messenger molecules diacylglycerol (DAG) and inositol 1,4,5-trisphosphate (IP3).  DAG mediates the activation of protein kinase C (PKC), while IP3 releases Ca(2+) from intracellular stores. |
| GNAQ | *guanine nucleotide binding protein (G protein), q polypeptide*  Guanine nucleotide-binding protein; modulates various transmembrane signaling systems |
| PTK2B | PTK2B protein tyrosine kinase 2 beta  Non-receptor protein tyrosine kinase; role in humoral immune response; regulates cell adhesion and migration |
| MAP3K5/  ASK1 | *mitogen-activated protein kinase kinase kinase 5 / Apoptosis signal-regulating kinase 1*  Serine/threonine protein kinase; essential component of MAP kinase signal transduction pathway; required for innate immune response against pathogens; important role in cellular responses to oxidative stress and changes in external environment |
| BCL10 | *B-cell CLL/lymphoma 10*  Promotes apoptosis and activation of NF-kappa-B  See Signaling pathways downstream of pattern-recognition receptors and their cross talk. Annu Rev Biochem 76:447-480 2007, Fig 1 and pg 462, and 2 |
|  | **Immunity-related signaling pathway** |
| TLR2 | *toll-like receptor 2*  Cell surface TLR, mediates innate immune response to bacterial lipoproteins and other cell wall components  See: Lee MS and Kim Y-J, Signaling pathways downstream of pattern-recoginition receptors and their cross talk. Annu Rev Biochem 76:447-480, 2007, Table 1.  El-Achkar, Renal Toll-like receptors: recent advances and implications for disease. Nat Clin Pract Nephrol, 2006: TLRs 1,2,3,4,5 and 7 were found to be more abundant than TLRs 6,8,9 and 10. Site of expression: see Table 1. |
| TLR3 | *toll-like receptor 3*  Cytoplasmic TLR, recognizes dsRNA viruses; interacts with adaptor molecule, TRIF, leading to NF-kappa-B activation  See TLR2 |
| TLR4 | *toll-like receptor 4*  Cell surface TLR, mediates innate immune response to bacterial lipopolysaccharide, leading to NF-kappa-B activation |
| TLR7 | *toll-like receptor 7*  Cytoplasmic TLR, recognizes ssRNA viruses; recruits MYD88 and activates NF-kappa-B |
| MAP3K14/  NIK | *mitogen-activated protein kinase kinase kinase 14 / NF-kappa-beta-inducing kinase*  Lymphotoxin beta-activated kinase, which seems to be exclusively involved in the activation of NF-kappa-B and its transcriptional activity.  Promotes proteolytic processing of NFKB2/P100, which leads to activation of NF-kappa-B *via* the non-canonical pathway. |
| MYD88 | *myeloid differentiation primary response gene (88)*  Adapter protein; involved in IL-1 and Toll-like receptor signaling; central role in innate and adaptive immune responses  Sequestered by IL1RL1 (ST2L)  See Lee MS, Signaling pathways downstream of pattern-recognition receptors and their cross talk. Ann Rev Biochem 2007 and Akira S, Pathogen Recognition and Innate Immunity. Cell 2006. |
| TRAF6 | *TNF receptor-associated factor 6*  E3 ubiquitin ligase; leads to NF-kappa-B activation  TNFAIP3 (A20) cleaves the ubiquitin chain of TRAF6, thus impairs IKK and p38/JNK signaling.  LY96 (β-arrestins) inhibits TRAF6 autoubiquitination and thus NFkB and AP-1 activation  See Akira S, Toll-like receptor signaling. Nat Rev Immunol, 2004 |
| IRAK4 | *interleukin-1 receptor-associated kinase 4*  Serine/threonine protein kinase; initiates innate immune response |
| IFNAR2 | *interferon (alpha, beta and omega) receptor 2*  Receptor for interferons alpha and beta. Involved in IFN-mediated JAK pathway, and STAT1, STAT2 and STAT3 activation. |
| IFNGR2 | *interferon gamma receptor 2 (interferon gamma transducer 1)*  This accessory factor is an integral part of the IFN-gamma signal transduction pathway and is likely to interact with GAF, JAK1, and/or JAK2 |
| FOS | *v-fos FBJ murine osteosarcoma viral oncogene homolog*  Dimerizes with proteins of the JUN family, thereby forming the transcription factor complex AP-1  Transcription factor AP-1 complex is composed of FOS and JUN, which are induced via MAP kinase cascade.  PKC activation (mainly β- and δ isoforms) resulted in c-fos and c-jun expressions, that combine in homo/heterodimers to form AP-1 transcription complex. This complex binds to, and induces expression of TGFb, fibronectin, laminin and ECM matrix proteins.  See PKC-βII |
| JUN | *jun oncogene*  See FOS  Combined with AP-1 (Fos/Jun complex) to regulate immune-related genes (Macian F, Partners in transcription: NFAT and AP-1, Oncogene 2001). |
| IRF3 | *interferon regulatory factor 3*  Mediates interferon-stimulated response element (ISRE) promoter activation. Functions as a molecular switch for antiviral activity. |
| IL18R1 | *interleukin 18 receptor 1*  Receptor for interleukin 18 (IL-18). Binding to the agonist leads to the activation of NF-kappa-B |
|  | **Negative regulators of signaling pathway** |
| TSLP | *thymic stromal lymphopoietin*  Epithelial cytokine; induces monocytes to release T cell-attracting chemokines  The most noticeable cytokine produced by epithelial cells and has major impact on Th1/Th2 immunity. TSLP activate DCs to favour Th2 induction.  Rimoldi, M., Chieppa, M., Salucci, V., Avogadri, F., Sonzogni, A., Sampietro, G. M., Nespoli, A., Viale, G., Allavena, P., Rescigno, M. (2005) Intestinal immune homeostasis is regulated by the crosstalk between epithelial cells and dendritic cells. Nat. Immunol. 6, 507–514.  Xu W, et al. (2007) Epithelial cells trigger frontline immunoglobulin class switching  through a pathway regulated by the inhibitor SLPI. Nat Immunol 8:294 –303.  Liu YJ, TSLP: an epithelial cell cytokine that regulates T cell differentiation by conditioning dendritic cell maturation. Annu Rev Immunol. 2007;25:193-219 |
| IL-10 | *Interleukin 10*  Inhibits synthesis of several cytokines e.g. IL-2, IL-3, IFN-γ |
| IL-10Rb | *interleukin 10 receptor, beta*  Cell surface receptor for activation of IL-10, IL-22, IL-26, IL-28 and IL-29 |
| IL-4 | *Interleukin 4*  Induces expression of class II MHC molecules on B cells; involved in several B –cell activation processes  IL4 is the major stimulus for the production of IgE antibodies by B cells, induces development of T_H_2 cells from naïve CD4^+^ helper T cells and inhibits differentiation of T_H_1 cells. |
| IL-1RN | *interleukin 1 receptor antagonist*  Inhibits interleukin-1 activity by binding to its receptor, IL1-R1 |
| IL13RA2 | *interleukin 13 receptor, alpha 2*  This protein binds IL13 with high affinity, but lacks cytoplasmic domain, and does not appear to function as a signal mediator. It is reported to play a role in the internalization of IL13 |
| TOLLIP | *toll interacting protein*  Ubiquitin-binding protein; involved in IL-1 and Toll-like receptor signaling. Inhibits cell activation by microbial products. Recruits IRAK1 to the IL-1 receptor complex. Inhibits IRAK1 phosphorylation and kinase activity. |
| ARRB1 | *arrestin, beta 1*  Regulates agonist-induced G-protein coupled receptor signaling by mediating both receptor desensitization and resensitization; involved in attenuation of NF-kappa-B-dependent transcription in response to GPCR or cytokine stimulation by interacting with and stabilizing CHUK  Involved in Toll-like receptor and IL-1 receptor signaling through the interaction with TRAF6, which prevents TRAF6 autoubiquitination and oligomerization required for activation of NF-kappa-B and JUN |
| SIGIRR | *single immunoglobulin and toll-interleukin 1 receptor (TIR) domain*  Negative regulator of Toll-like and IL-1R receptor signaling |
| IL1RL1/  ST2L | *interleukin 1 receptor-like 1, ST2 protein*  sequesters MyD88 and TIRAP |
| RNF216/  Triad3A | *ring finger protein 216 / Triad domain-containing protein 3*  Down-regulates NF-kappa-B and IRF3 activation, and IFN-β production; regulates antiviral responses.  Isoform 3/ZIN inhibits TNF and IL-1 mediated activation of NF-kappa-B. |
| SOCS1 | *suppressor of cytokine signaling 1*  Part of negative feedback system regulating cytokine signal transduction  See O'Sullivan, Cytokine receptor signaling thru Jak-Stat-Socs pathway in disease. Mol Immunol 2007.  SOCS are rapidly induced following cytokine stimulation (STAT-dependent) and are promptly degraded on cessation of signaling. SOCS1 and SOCS3 are induced by diverse mechanisms in macrophages in response to microbial products and may be responsible for suppressing JAK/STAT signaling. Thus SOCS proteins not only provide a mechanism for the innate immune system to prevent an excessive response to pathogenic challenge, but may also inhibit macrophage function during chronic antigen exposure. |
| NFKBIA/  IκBα | *Nuclear factor of kappa light polypeptide gene enhancer in B-cells inhibitor, alpha*  Inhibits NF-kappa-B/REL complexes involved in inflammatory responses.  See Lee MS, Signaling pathways downstream of pattern-recognition receptors and their cross talk. Ann Rev Biochem 2007 and Akira S, Pathogen Recognition and Innate Immunity. Cell 2006 |
| TNFAIP3/  A20 | *tumor necrosis factor, alpha-induced protein 3*  Ubiquitin-editing enzyme; ensures transient nature of inflammatory signaling.  Cleaves the ubiquitin chain of TRAF6, thus impairs IKK and p38/JNK signaling. |
| IRAK3/  IRAK-M | *interleukin-1 receptor-associated kinase 3*  Inhibits dissociation of IRAK1 and IRAK4 from the Toll-like receptor signaling complex |
| PIN1 | *peptidylprolyl cis/trans isomerase, NIMA-interacting 1*  Peptidyl-prolyl cis/trans isomerase; regulates immune response  PIN1 binds to the activated IRF3, causing its ubiquitination and subsequent proteasomal degradation. |
| PIAS1 | *protein inhibitor of activated STAT, 1*  Functions as an E3-type small ubiquitin-like modifier (SUMO) ligase, stabilizing the interaction between UBE2I and the substrate, and as a SUMO-tethering factor  See Liu B, Inhibition of Stat1-mediated gene activation by PIAS 1. PNAS 95: 10626-10631, 1998). |
| PTEN | *phosphatase and tensin homolog*  Dual specificity protein and lipid phosphatase; antagonizes PI3K-AKT signaling |
| UBA1 | *ubiquitin-like modifier activating enzyme 1*  Activates ubiquitin by adenylation with ATP |
|  | **Pathogen-associated molecular pattern (PAMP) receptors** |
| NR3C1 | *nuclear receptor subfamily 3, group C, member 1 (glucocorticoid receptor)*  One of the C-type lectins that recognizes microbial products including virus. It is also a receptor for glucocorticoids. Has a dual mode of action: as a transcription factor that binds to glucocorticoid response elements and as a modulator of other transcription factors. Affects inflammatory responses, cellular proliferation and differentiation in target tissues. Could act as a coactivator for STAT5-dependent transcription upon growth hormone stimulation. |
| HMGB1 | *high-mobility group box 1*  DNA-binding protein; preference for single-stranded DNA; associates with chromatin; able to bend DNA  HMGB proteins 1, 2, and 3 bind to all immunogenic nucleic acid and induce type I interferon and inflammatory cytokine. Defect in HMGB proteins showed impaired activation of TLR3, 7 and 9, IRF3 and NFkB (see Yanai H, HMGB proteins function as universal sentinels for nucleic-acid-mediated innate immune responses, Nature, 2009). |
| NLRP3/  NALP3 | *NLR family, pyrin domain containing 3*  Pyrin-like protein; contains a nucleotide-binding domain; regulates inflammation, immune response and apoptosis  Inhibits TNF-alpha induced activation and nuclear translocation of RELA/NF-KB p65. Also inhibits transcriptional activity of RELA. |
| NOD1 | *nucleotide-binding oligomerization domain containing 1*  Cytosolic protein with a nucleotide-binding domain; initiates inflammation in response to bacterial lipopolysaccharides; enhances caspase-9 mediated apoptosis |
| RARRES3/  RIG-1 | *retinoic acid receptor responder (tazarotene induced) 3*  RNA helicase; preferentially recognizes 5’-triphosphate viral RNA  RARRES3 (RIG1) and IFIH1 (MDA5) are RNA-sensing receptors in the cytosol. |
| TLR3 | *Toll-like receptor 3*  Cytoplasmic TLR; recognizes dsRNA viruses; interacts with adaptor molecule, TRIF, leading to NF-kappa-B activation |
| TLR7 | *Toll-like receptor 7*  Cytoplasmic TLR; recognizes ssRNA viruses; recruits MYD88 and activates NF-kappa-B |
|  | **Antimicrobial activity** |
| DEFB1 | *defensin, beta 1*  Bactericidal activity |
| LEAP2 | *liver expressed antimicrobial peptide 2*  Antimicrobial activity |
| LTF | *Lactotransferrin*  Iron-binding protein with antimicrobial activity; important component of the non-specific immune system |
| PIGR | *polymeric immunoglobulin receptor*  A member of the immunoglobulin superfamily; expressed on several glandular epithelia and plays a crucial role in the mucosal immune defence; mediates transcellular transport of polymeric immunoglobulin molecules; expression increases in response to interferon-gamma, IL-4, IL-1 and TNFa (MAPK- and PI3K-mediated, and negatively-regulated by ERK pathway)  See Takenouchi-Ohkubo, Immunol 123: 500-507, 2008 |
|  | **Complement system and adaptive immunity** |
| C2 | *complement component 2*  Part of the classical complement system; cleaved by activated C1 to a serine protease |
| C3 | *complement component 3*  Central role in both classical and alternative complement pathways |
| C4A/4B | *complement component 4A (Rodgers blood group) / complement component 4B (Chido blood group)*  Central role in activation of classical complement pathway |
| C5 | *Complement component 5*  Involved in assembly of C5-C9 into the membrane attack complex |
| TAP1 | *transporter 1, ATP-binding cassette, sub-family B (MDR/TAP)*  Antigen presentation; transports antigens to endoplasmic reticulum for association with class I MHC molecules.  See Klein J, The HLA system. NEJM, 2000: The most dramatic malfunction of the HLA system occurs when its genes falter in their expression, resulting in the bare lymphocyte syndrome – immunodeficiency syndrome.  HLA class I deficiency is caused by a defect in the TAP genes (TAP1, TAP2 and TAPBP) |
| TAP2 | *transporter 2, ATP-binding cassette, sub-family B (MDR/TAP)*  Antigen presentation; transports antigens to endoplasmic reticulum for association with class I MHC molecules. |
| TAPBP | *TAP binding protein (tapasin)*  Antigen presentation; involved in association of class I MHC molecules with TAP, and in peptide loading |
| HLA-A | *major histocompatibility complex, class I, A*  Class I MHC molecule; presents peptides to CD8^+^ T cells  Class I MHC pathway: engage by CD8^+^ T cells, serves to eradicate infections by intracellular microbes that reside in the cytoplasm of infected cells  See Abbas, Cellular and Molecular Immunology text book, table 6-3, pg 124 |
| HLA-E | *major histocompatibility complex, class I, E*  Class I MHC molecule; presents peptides to CD8^+^ T cells  See HLA-A |
| HLA-DPA1 | *major histocompatibility complex, class II, DP alpha 1*  Class II MHC molecule; presents peptides to CD4^+^ T cells  Class II MHC pathway: engage by CD4^+^ T cells (cytokine-producing helper T cells) function in host defense against extracellular microbes; high expression suggests increased microbial threat. |
| HLA-DQA1 | *major histocompatibility complex, class II, DQ alpha 1*  Class II MHC molecule; presents peptides to CD4^+^ T cells  See HLA-DPA1 |
|  | **Antioxidants and oxidants** |
| NFE2L2/  Nrf2 | *nuclear factor (erythroid-derived 2)-like 2*  Transcription activator; binds antioxidant response elements in promoters of target genes; co-ordinates upregulation of genes in response to oxidative stress  Huang HC, Regulation of the antioxidant response element by protein kinase C-mediated phosphorylation of NF-E2-related factor 2. PNAS 97: 12475-80, 2000: PKC-directed phosphorylation of Nrf2 may be a critical event for the nuclear translocation of this transcription factor in response to oxidative stress.  Salazar M, Glycogen synthase kinase-3beta inhibits the xenobiotic and antioxidant cell response by direct phosphorylation and nuclear exclusion of the transcription factor Nrf2. JBC, 2006: Nrf2 regulates the expression of antioxidant phase II genes and contributes to preserve redox homeostasis and cell viability in response to oxidant insults. Nrf2 up-regulated the expression of HO-1, glutathione peroxidase, glutathione S-transferase A1, NAD(P)H: quinone oxidoreductase and glutamate-cysteine ligase and protected against hydrogen peroxide-induced glutathione depletion and cell death |
| SOD2 | *superoxide dismutase 2, mitochondrial*  Degrades superoxide anion radicals to hydrogen peroxide, which is then converted to H2O and O_2_ by other enzymes, e.g. catalase  See: Carl Nathan, Reactive oxygen and nitrogen intermediates in the relationship between mammalian hosts and microbial pathogens, PNAS 2000 |
| CAT | *Catalase*  Converts hydrogen peroxide to water; mitigates oxidative stress |
| GPX3 | *glutathione peroxidase 3*  Catalyzes reduction of hydrogen peroxide, lipid peroxides by glutathione |
| GSS | *glutathione synthetase*  Catalyzes glutathione biosynthesis; protects against oxidative damage by free radicals |
| HMOX1 | *heme oxygenase (decycling) 1*  Cleaves heme to biliverdin and catalyzes heme breakdown to release iron, carbon monoxide; products have potent antioxidant action |
| MSRA | *methionine sulfoxide reductase A*  Repair enzyme for proteins inactivated by oxidation |
| NOS3 | *nitric oxide synthase 3 (endothelial cell) (eNOS)*  Expression of endothelial nitric oxide synthetase (eNOS) is repressed by activation of PKC-β. eNOS is an important antiatherogenic enzyme that prevents vascular pathology |
| NOX4 | *NADPH oxidase 4*  Renal isoform of NADPH oxidase; generates superoxide intracellularly |
| NQO1 | *NAD(P)H dehydrogenase, quinone 1*  Quinone reductase; prevents production of oxygen radical species  Rushworth SA, Lipopolysaccharide-induced expression of NAD(P)H:quinone oxidoreductase 1 and heme oxygenase-1 protects against excessive inflammatory responses in human monocytes. J Immunol, 2008: Silencing expression of NQO1 alone, or in combination with heme oxygenase-1 (HO-1) silencing, markedly increased LPS-induced TNF and IL-1beta expression. |
| TXNRD1 | *thioredoxin reductase 1*  Has glutaredoxin and thioredoxin reductase activities; protects against oxidative stress. |

* Information on gene/protein functions was extracted from www.genecards.org and [www.ncbi.nlm.nih.gov](http://www.ncbi.nlm.nih.gov).

General reference:

1. Brownlee M (2005) The pathobiology of diabetic complications. A unifying mechanism. Diabetes 54: 1615-1625.
2. Akira S, Uematsu S, Takeuchi O (2006) Pathogen recognition and innate immunity. Cell 124: 783-801.
3. Swamy M, Jamora C, Havran W, Hayday A (2010) Epithelial decision makers: in search of the epimmunome. Nat Immunol 11: 656-665.
4. Li MO, Wan YY, Sanjabi S, Robertson AL, Flavell RA (2006) Transforming growth factor-β regulation of immune responses. Annu Rev Immunol 24: 99-146.
5. H Noh, GL King (2007) The role of protein kinase C activation in diabetic nephropathy. Kidney Int 72: 549-553.
