# Supplementary figures and images for: Negative regulatory responses to metabolically triggered inflammation impair renal epithelial immunity in diabetes mellitus
Source: J Mol Med (Berl). 2012 Nov 14;91(5):587–98. doi: 10.1007/s00109-012-0969-x (PMC3644409; doi:10.1007/s00109-012-0969-x)

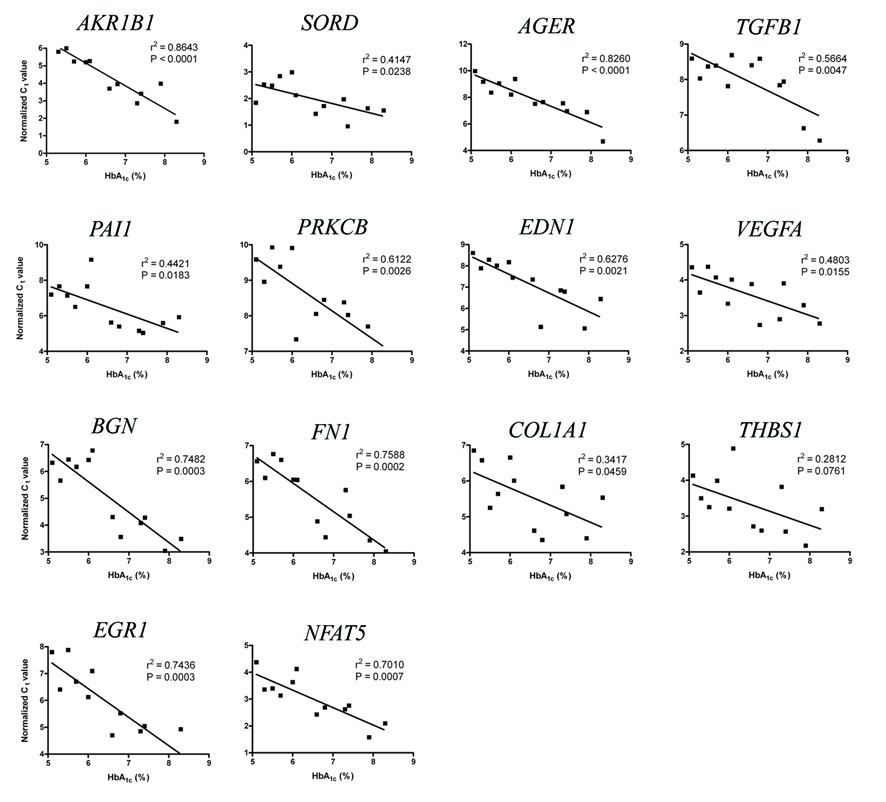

Supplement: Supplementary file 2 — (GIF 1.87 MB) [file 109_2012_969_Fig1_ESM.jpg]

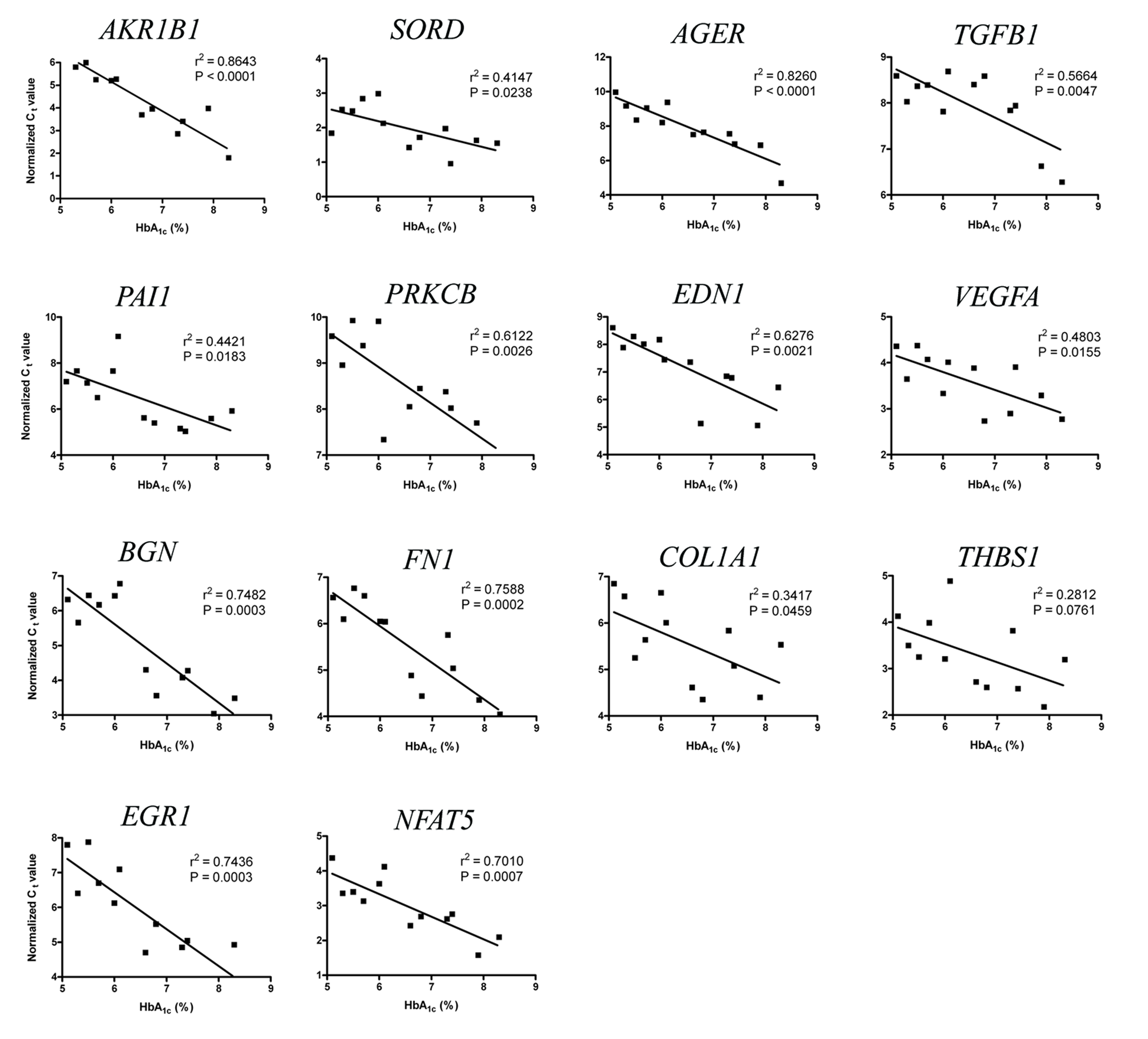

Supplement: Supplementary file 3 — High resolution image file (TIF 1.23 MB) [file 109_2012_969_Fig1_ESM.tif]
